# Supplementary material for: Multi-proton dynamics near membrane-water interface
Source: Nat Commun. 2025 Apr 6;16:3276. doi: 10.1038/s41467-025-58167-w (PMC11972326; doi:10.1038/s41467-025-58167-w)
Supplement: Supplementary file 1 — Supplementary Information [file 41467_2025_58167_MOESM1_ESM.pdf]

Supplementary Information for

Multi-Proton Dynamics near Membrane-Water Interface

Subhasish Mallick[1] and Noam Agmon[1]*

[1]The Fritz Haber Research Center, Institute of Chemistry, The Hebrew University of Jerusalem, Jerusalem 9190401, Israel

E-mail: agmon@fh.huji.ac.il

Contents:

No.	Title	Page No.
Supplementary Table 1	Details of the equilibration steps for the classical trajectory of lipid plus water, reducing the positional and dihedral restraining force constants (FC) in each step.	S3
Supplementary Figure 1	The time-dependence of the mobile proton mean squared displacement (MSD) using the single origin averaging method for (a) H_a^+ in simulation 1b, (b) H_b^+ in simulation 2a, (c) H_b^+ in simulation 2b, and (d) H_c^+ in simulation 3a. Compare with Figures 3d, 4c, 4f, and 5c in the main text, where multiple-origin averaging was used.	S3
Supplementary Figure 2	The time-dependent MSD was obtained using DFTB3 for an excess proton in pure water (no lipids) up to 10 ps. The red dotted line depicts a linear fit using MSD(t) = $6Dt$, with D = 0.86 Å^2/ps (see Table 1).	S4
Supplementary Figure 3	Motion perpendicular to the lipid bilayer, $Z_P(t)$, of three (out of 8) phosphate atoms in the upper leaflet of Simulation 3a, as compared to the average of all 8 ($\langle Z_P \rangle$, red line). Clearly, this simple averaging procedure reduces substantially the random fluctuations in $Z(t)$. However, it is unclear why $\langle Z_P \rangle$ increases with time.	S4
Supplementary Figure 4	Temporal evolution, during 2 ps, of (a) the kinetic energy (in blue) and (b) the potential energy (in red) for our system, having 16 POPC lipids and 458 water molecules (without any proton), using DFTB3 to propagate the coordinates of the pre-equilibrated	S5

	classical trajectory. The progression of these energies suggests that the system reached its equilibrium state within 2 ps. This rapid equilibration is understood because the system was thoroughly equilibrated classically in GROMACS. Other degrees of freedom may equilibrate on slower timescales.	
Supplementary Figure 5	The time-dependent MSD calculated for free Na^+ (i.e., not directly interacting with the lipids) within a system composed of 8+8 POPC lipids, 477 water molecules, 3 Na^+, and 3 Cl^- ions up to 20 ps. The red dotted line depicts a linear fit using MSD(t) = 6Dt, with D = 0.057 Å^2/ps.	S5
Supplementary Figure 6	Distance of (a) H_a^+ and (b) H_b^+ from the nearest phosphatic oxygen (O_P) in the lower leaflet of our BOMD Simulation 2a’, using the BLYP-D3 functional in the QUICKSTEP module of CP2K. H_a^+ is covalently bound (at a distance of 1 Å) for the duration of the simulation. H_b^+ fluctuates between the bound state and (at least) two hydrogen-bonded states.	S6
Supplementary Figure 7	Oxygen-oxygen radial distribution functions for bulk water treated with the diagonal DFTB3 method (present work), and compared with experiment, see Soper, A. K.; Benmore, C. J. Quantum differences between heavy and light water. *Phys. Rev. Lett.* **101**, 065502 (2008).	S6

Supplementary Table 1: Details of the equilibration steps for the classical trajectory of lipid plus water, reducing the positional and dihedral restraining force constants (FC) in each step.

Step No.	Simulation Time (ps)	Time step (fs)	Positional FC (kJ $mol^{-1}nm^{-2}$)	Dihedral FC (kJ mol^{-1} rad^{-2})
1	125	1.0	1000	1000
2	125	1.0	400	400
3	250	1.0	400	200
4	250	2.0	200	200
5	250	2.0	40	100
6	1000	2.0	None	None

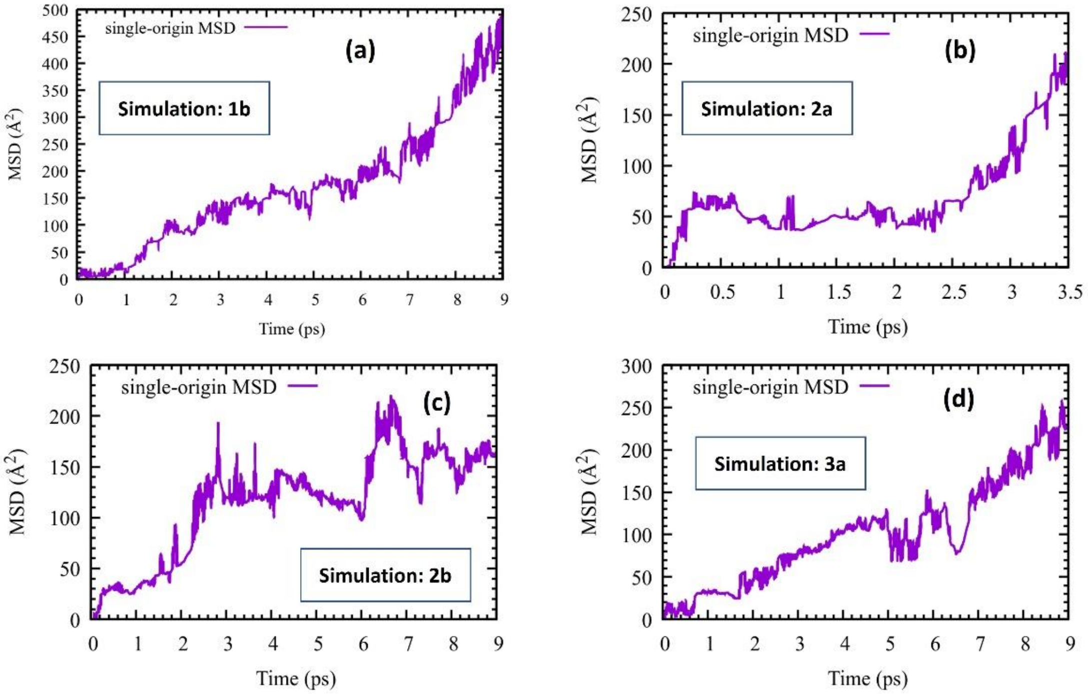

Supplementary Figure 1: The time-dependence of the mobile proton mean squared displacement (MSD) using the single origin averaging method for (a) H_a^+ in simulation 1b, (b) H_b^+ in simulation 2a, (c) H_b^+ in simulation 2b, and (d) H_c^+ in simulation 3a. Compare with Figures 3d, 4c, 4f, 5c and 5f in the main text, where multiple-origin averaging was used.

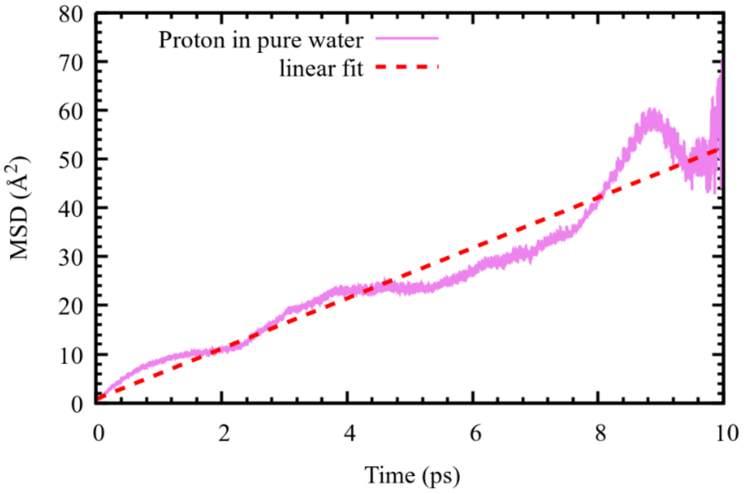

Supplementary Figure 2: The time-dependent MSD obtained using DFTB3-diag for an excess proton in pure water (no lipids) up to 10 ps. The red dotted line depicts a linear fit to MSD(t) = 6Dt, with D = 0.86 Å^2/ps (last line in Table 1 in main text).

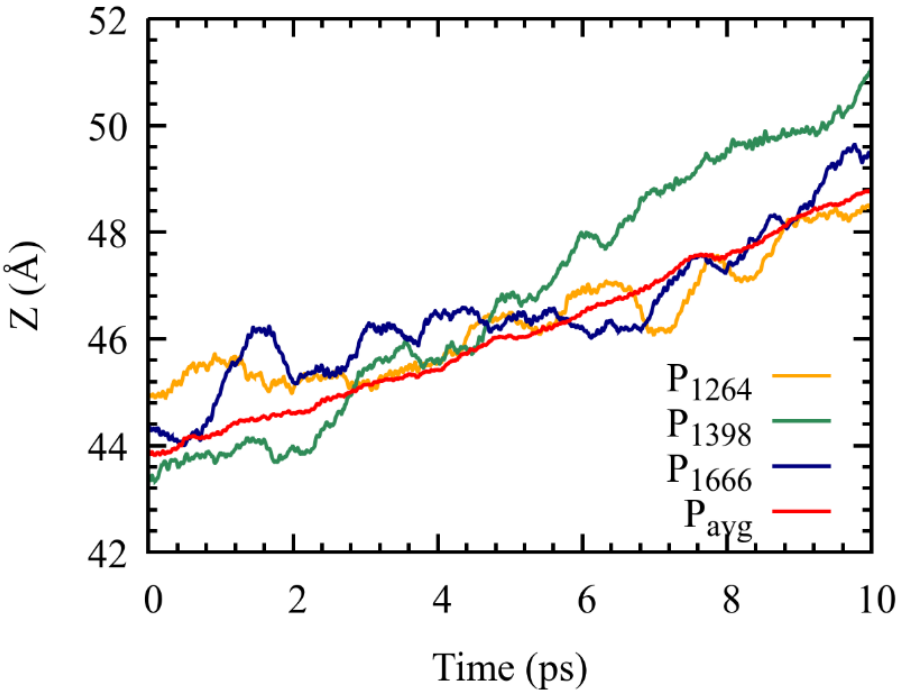

Supplementary Figure 3: Motion perpendicular to the lipid bilayer phosphate plane, $Z_P(t)$, of three (out of 8) phosphate atoms in the upper leaflet of Simulation 3a, as compared to the average of all 8 ($\langle Z_P \rangle$, red line). Clearly, this simple averaging procedure reduces substantially the random fluctuations in $Z(t)$. However, it is unclear why $\langle Z_P \rangle$ increases with time.

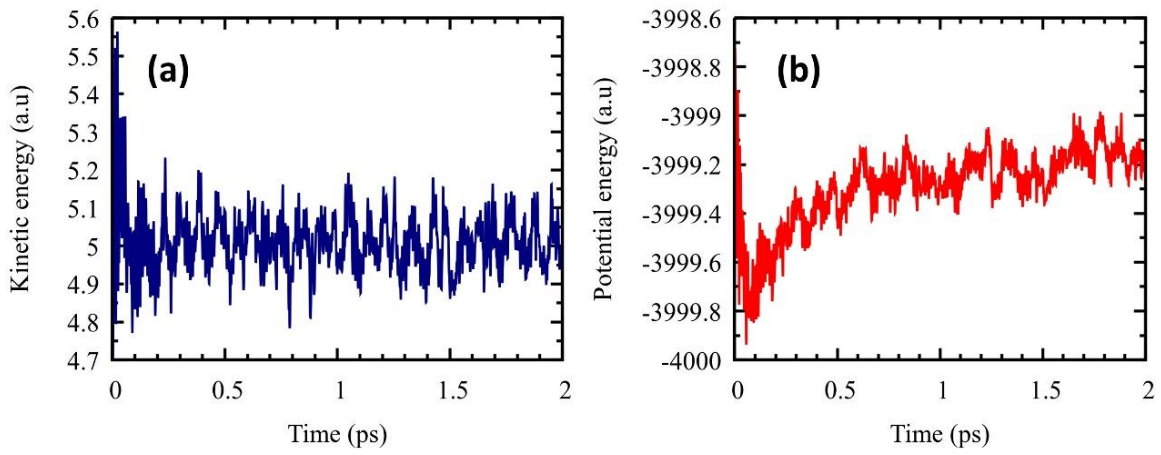

Supplementary Figure 4: Temporal evolution, during 2 ps, of (a) the kinetic energy (in blue) and (b) the potential energy (in red) for our system, consisting of 16 POPC lipids and 458 water molecules (but without any proton), using DFTB3-diag to propagate the coordinates of the pre-equilibrated classical trajectory. The progression of these energies suggests that the system reached its equilibrium state within 2 ps. This rapid equilibration is understood because the system was already thoroughly equilibrated classically in GROMACS. Other degrees of freedom may equilibrate on slower timescales.

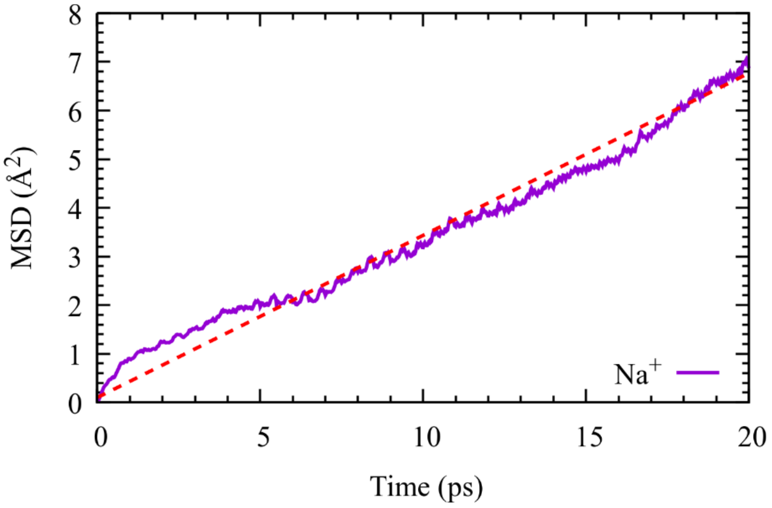

Supplementary Figure 5: The time-dependent MSD calculated for free Na^+ (i.e., not directly interacting with the lipids) within a system composed of 8+8 POPC lipids, 477 water molecules, 3 Na^+, and 3 Cl^- ions propagated up to 20 ps at 300 K. The red dotted line depicts a linear fit using MSD(t) = 6Dt, with D = 0.057 Å^2/ps during the first 100 ps. It becomes 0.061 Å^2/ps during the first 2 ns. In comparison, for sodium in pure water with 400 mM NaCl we have calculated D = 0.096 Å^2/ps. As might be expected, this is ca. 50 % larger than sodium in the water phase above a POPC membrane. The behavior of the proton is thus anomalous, speeding up near the membrane.

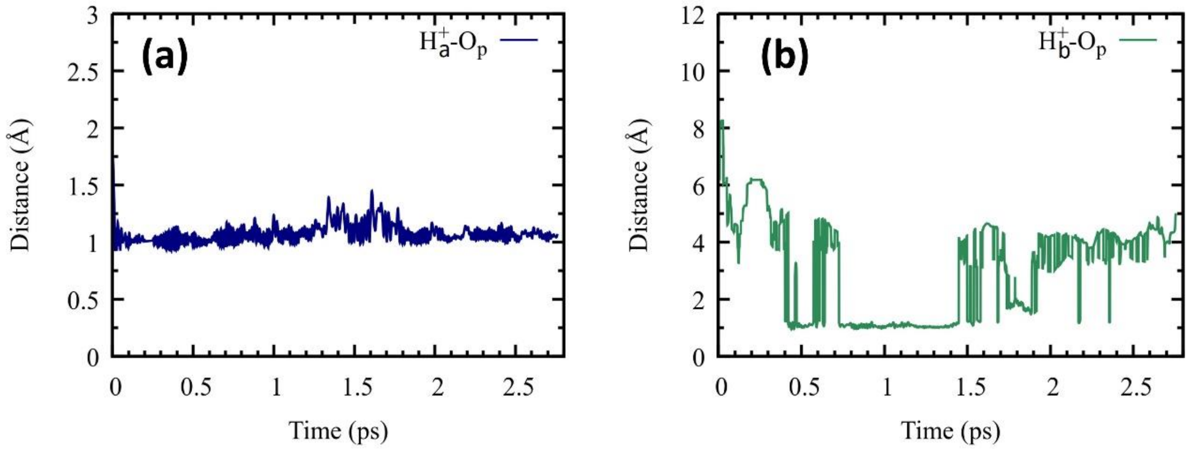

Supplementary Figure 6: Distance of (a) H_a^+ and (b) H_b^+ from the nearest phosphatic oxygen (O_P) in the lower leaflet of our BOMD Simulation 2a', using the BLYP-D3 functional in the QUICKSTEP module of CP2K. H_a^+ is covalently bound (at a distance of 1 Å) for the duration of the simulation. H_b^+ fluctuates between the bound state and (at least) two hydrogen-bonded states.

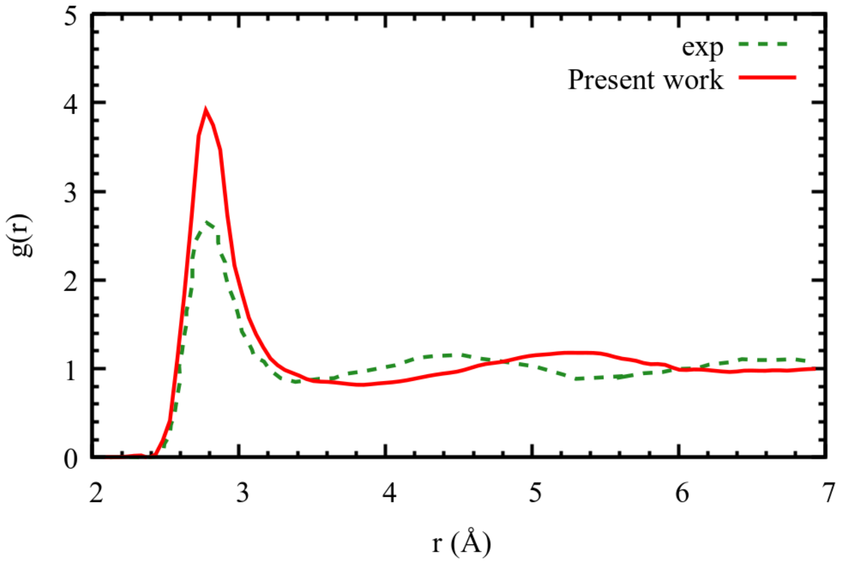

Supplementary Figure 7: Oxygen-oxygen radial distribution functions for bulk water treated with the diagonal DFTB3 method (present work), and compared with experiment, see Soper, A. K.; Benmore, C. J. Quantum differences between heavy and light water. *Phys. Rev. Lett.* **101**, 065502 (2008).
