# Supplementary material for: Multi-proton dynamics near membrane-water interface
Source: Nat Commun. 2025 Apr 6;16:3276. doi: 10.1038/s41467-025-58167-w (PMC11972326; doi:10.1038/s41467-025-58167-w)
Supplement: Supplementary file 2 — Description of Additional Supplementary Files [file 41467_2025_58167_MOESM2_ESM.pdf]

Description of Additional Supplementary Files:

Supplementary Data 1: Initial and final configurations. The XYZ coordinates for the initial and final configurations of all DFTB3 trajectories.

Supplementary Movie 1: Simulation 1a: The proton (H_a^+) forms a covalent bond instantly with its nearest phosphatic oxygen (O_P). However, as H_a^+ was likely not yet equilibrated, it promptly (within 400 fs) escaped from the membrane surface into the bulk. It then engaged in lateral diffusion parallel to the membrane for approximately 2 ps before abruptly approaching the membrane again via a water wire, protonating another phosphate group.

Supplementary Movie 2: Simulation 1b: H_a^+ was initially placed in three hydration layers from the nearest O_P. Subsequently, H_a^+ moved along the Z-axis for 2 ps toward the upper leaflet before suddenly reverting and returning to the bulk.

Supplementary Movie 3: Simulation 2a: H_a^+, placed near the lower leaflet, instantly engaged in a covalent bond with the nearest O_P. Meanwhile, H_b^+ that was initially positioned away from the membrane migrated toward the bulk region, reaching the middle of the water layer. It subsequently diffused parallel to the membrane for about 2.2 ps. Then, it altered its course, veering toward the upper leaflet, inducing the formation of a water wire with an O_P atom, which rapidly became protonated.

Supplementary Movie 4: Simulation 2b: H_a^+ is already attached to a phosphate group in the lower leaflet. Meanwhile, H_b^+ exhibited significant lateral mobility in the bulk region.

Supplementary Movie 5: Simulation 3a: H_a^+, and H_b^+, initially placed near the lower and upp er membrane surface, formed covalent bonds with the nearest O_P, while H_c^+ moved laterally.

Supplementary Movie 6: Simulation 3b: H_a^+, and H_b^+, initially positioned near the lower and upper membrane surfaces respectively, quickly formed covalent bonds with the nearest O_P. In contrast, H_c^+ diffused parallel to the membrane surface, remaining close to it rather than moving into the bulk water phase.

Supplementary Movie 7: Simulation Na: Three sodium ions were initially placed randomly within the system. Among them, one formed a non-covalent interaction with the lipid head group, while the others diffused freely in the water phase.
